# Ocular involvement in metastatic and systemic malignancies is not rare

**DOI:** 10.1002/cnr2.1347

**Published:** 2021-02-03

**Authors:** Purnima Rajkarnikar Sthapit, Rohit Saiju

**Affiliations:** ^1^ Department of Ocular Oncology and Oculoplasty Tilganga Institute of Ophthalomology Kathmandu Nepal

**Keywords:** choroidal metastasis, eye cancer, ocular malignancy, ocular metastasis, orbital metastasis

## Abstract

**Background:**

Metastatic disease to the eye most commonly involves choroid followed by orbit leading to varied ocular manifestations. By comparison, it is relatively rarer than primary malignancies of eye as well as metastasis in other parts of the body.

**Aim:**

The aim of this study is to evaluate the common eye and orbital structures involved in secondary ocular and metastatic disease, to describe its clinical manifestations and outline the management done.

**Methods:**

A retrospective study of newly diagnosed cases of ocular metastasis in last 2 years conducted in our recently established department of ocular oncology at a tertiary eye care hospital in Nepal. Demography, age and sex distribution were noted. The patients were segregated into those with secondary or metastatic ocular malignancies. Detail study on the metastatic disease to eye was made in regards to presenting symptoms, signs, primary site of cancer, and the treatment done. Details of the investigations done, like biopsy and imaging were also recorded.

**Results:**

There were a total of 28 patients, whose age group ranged from 9 years to 69 years with median age of 43 years. Females constituted 46% of total patients. Both the eyes were involved in 9 patients (32%). Eye was secondarily involved by paranasal sinus tumors and Non Hodgkin lymphoma (7 patients each). Ocular metastasis was commonly seen from broncogenic carcinoma in four and breast carcinoma in three patients. Simultaneous metastasis to other parts of the body was also seen in 61% of our patients. Diminution of vision in 49% was the most common presenting feature followed by proptosis in 16% and palpable mass in 14% of patients. Orbit in 43% cases is the commonest ocular structure involved. Histopathologic diagnosis was done in 32% only while rest was based on imaging alone. The most common treatment done was chemotherapy in 57% patients.

**Conclusion:**

Ocular metastasis can display a wide variety of clinical and imaging features and therefore a high degree of suspicion is required. It is often associated with simultaneous metastasis to other parts of the body as well, hence the importance of earlier diagnosis and metastatic workup.

## 1 INTRODUCTION

Secondary ocular malignancies are not uncommon. Ocular malignancy is the least discussed topic in oncology forum; much less is reported in oncology journals. However the scenario is more griever that it appears. Ocular oncologists and other ophthalmologists frequently encounter lesions suspicious of eye cancer; most are primary malignancies arising from different parts of eye and orbit within the age group ranging from neonates to old age. In children retinoblastoma is common primary malignancy and in adults, squamous cell carcinoma of conjunctiva, melanoma, and adenocarcinomas are common. However secondary malignancies from adjacent structures like paranasal sinuses, infiltration from systemic cancers like leukemia, and even distant metastasis from other parts of the body are also frequently encountered by ocular oncologist.

Metastatic disease to the eye most commonly involves the choroid followed by orbit.^1^ By comparison, it is relatively uncommon accounting for less than 5%^2^of all orbital tumors and occurring in about 7% of all cancers of body.^3^ The common primary sites in adults being lung in males and breast in females and they account for 50% of all orbital metastases.^4^ In approximately 20% of patients however, orbital metastasis is the initial manifestation of systemic malignancy.^5^ Therefore, ocular manifestation of systemic malignancies should be known to both medical oncologist as well as ophthalmologist.

However, due to infrequent nature of carcinomas metastatic to orbit, most articles are in the form of case report rather than research papers. Our objective is to evaluate and describe the clinical manifestations and its management of secondary as well as metastatic carcinomas invading the eye and orbital structures.

### 1.1 Methodology

This is a retrospective, noncomparative, and descriptive study conducted from October 2017 to September 2019 in the department of ocular oncology at a tertiary care eye hospital in Nepal. Institutional review board approval has been obtained. The data was collected from our comprehensive electronic medical record files. Clinical photographs, fundus photographs, and imaging films were collected where indicated, after obtaining a written informed consent from the patients.

Demographic data recorded included age, gender, race, laterality, and eye involved. The presenting ocular symptoms and positive finding on detail examination of eye was recorded. Best corrected visual acuity at presentation was noted. History of presenting illness if known like, malignancy elsewhere in body was noted. Intraocular distribution of metastatic tumors was also studied. All the patients were also seen by a medical oncologist. Diagnosis and investigations obtained from their records as well. The patients were segregated into those with primary malignancy, invasion from adjacent structures outside orbit, infiltration from systemic cancers, and ocular metastasis from distant tumors. Primary ocular malignancies were excluded and the rest were included in the study. Diagnosis was based on histopathology wherever possible; if not it was made from detailed ocular examination and imaging like CT scan and MRI. If the patient was a known case of carcinoma elsewhere, it was taken as a distant metastasis based on imaging alone.

The clinical data were recorded and analyzed with regards to common age group affected by secondary tumors; it is presenting clinical features, anatomic site of eye involved, vision affected, and common primary malignancy secondarily affecting the eye. Treatment done in the form of medical, surgical or both was also recorded.

## 2 RESULTS

A total of 28 patients with secondary ocular malignancy were enrolled in the study. Their age group ranged from 9 years to 69 years with mean age of 42.6 years mode of 50 years median age being 43 years. Females constituted 46% of patients and rest of 54% were males. Both the eyes were involved in nine patients (32%), rest had unilateral involvement. Diminution of vision in 13 patients (49%) was the most common presenting feature. Four patients (11%) had NPL (no perception of light which is equivalent to complete loss of vision) while it was less than 3/60 in 14 patients (38%) as shown in Table 1. Next common presenting complain was proptosis (forward protrusion of eyeball) in four patients (16%). Rest of the presenting clinical features are shown in Figure  2.

**TABLE 1:** Best corrected visual acuity

Vision	No of eyes (n‐37)	Percentage (%)
<3/60	14	37.8
3/60‐5/60	2	5
6/60‐6/18	5	13.5
6/12‐6/6	12	32.4
NPL^a^	4	10.8

^a^ No perception of light.

**FIGURE 1:**
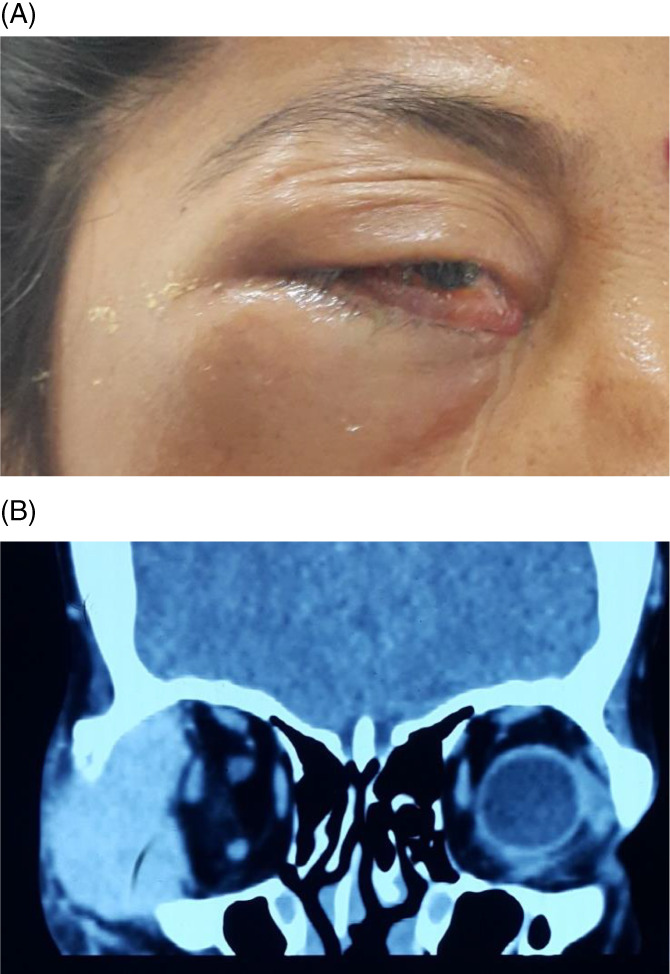
Breast carcinoma metastasizing to orbit and temporal fossa

Table 2 highlights the various types of secondary and metastatic cancers involving eyeball and orbit. In seven patients, sinonasal carcinoma produced local invasion to the adjacent orbit presenting as proptosis as seen in Figure 4. Non Hodgkin lymphoma was the commonest hamatogenic malignancy leading to infiltration in eye (Figure 5), however other less common ones were multiple myeloma and acute lymphoblastic leukemia. Apart from above, metastasis to eye from distant primary sites are also seen, the common ones being from bronchogenic carcinoma in male and breast carcinoma in female (Figure 1).

**TABLE 2:** Types of secondary and metastatic cancers involving eye and orbit

Type of secondary tumor	Primary malignancy	No of patients (n = 28)	Percentage (%)
Local invasion	Sinonasal carcinoma	7	25
Infiltration from hamatogenic cancers	Non Hodgkin lymphoma	7	25
	Acute lymphoblastic lymphoma	2	7
	Multiple myeloma	1	3.5
Distant metastasis	Broncogenic carcinoma	4	14.2
	Breast carcinoma	3	10.7
	Choriocarcinoma	1	3.5
	Rectal carcinoma	1	3.5
	Oesophageal carcinoma	1	3.5
	Prostrate carcinoma	1	3.5

**FIGURE 2:**
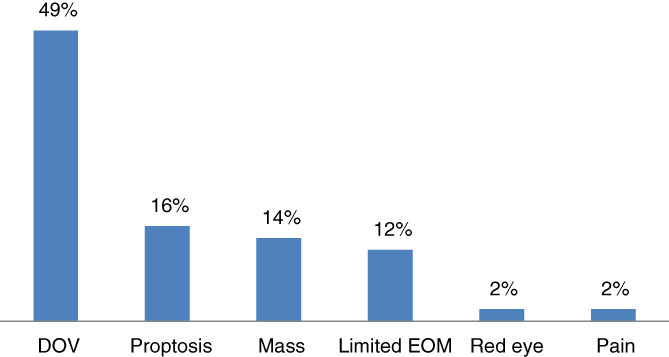
Clinical features at presentation

**FIGURE 3:**
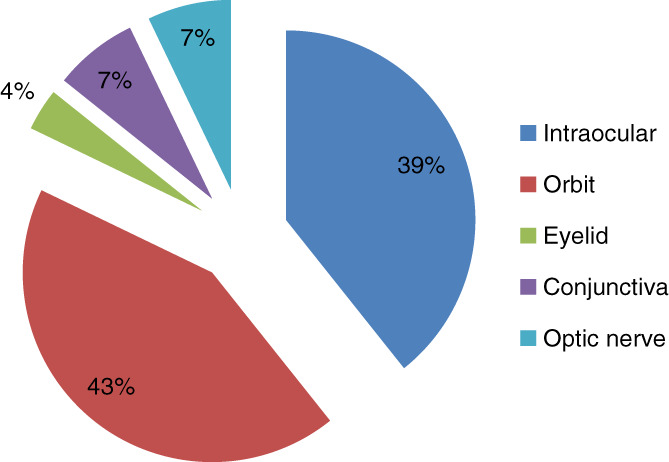
Ocular structures involved in secondary tumors

Secondary malignancy in the form of orbital space occupying lesion was the most common presentation as shown in Figure 3. Intraocular infiltration in choroid and retina was next common, in 39% of patient.

**FIGURE 4:**
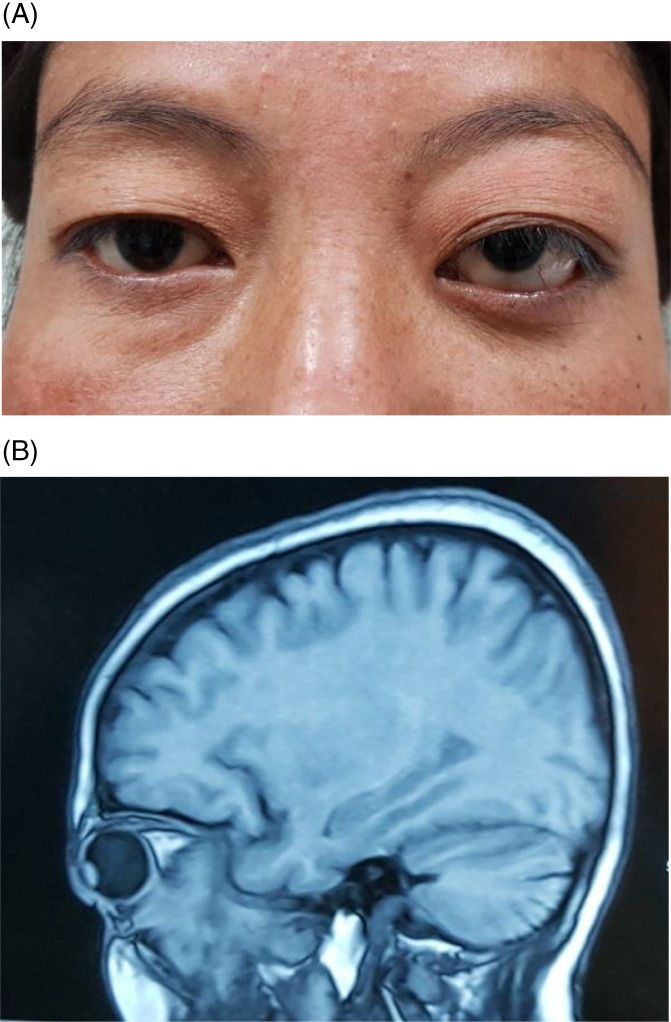
Sinonasal carcinoma invading left orbit. (A) Presenting as proptosis. (B) MRI image showing maxillary carcinoma invading the floor and retrobulbar orbit

**FIGURE 5:**
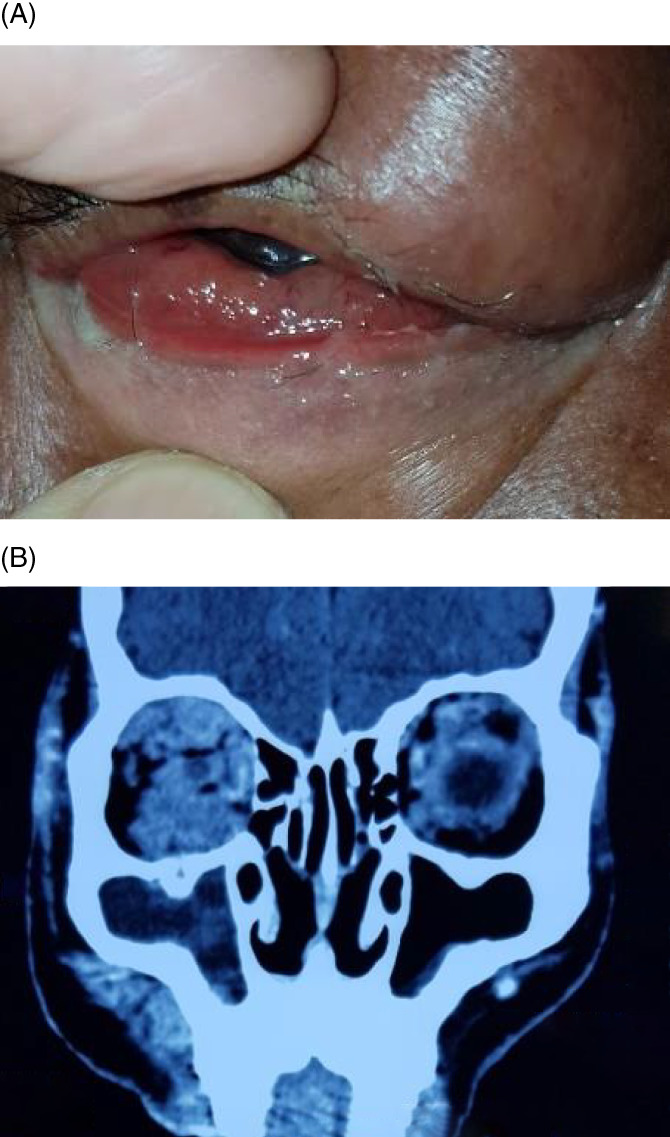
Orbital infiltration of Non‐Hodgkin lymphoma. (A) Conjunctival and orbital lymphoma. (B) CT scan image showing bilateral diffuse orbital infiltration

The patients were managed with chemotherapy alone in 57% and combined with surgery in 14% of cases. Surgery alone was done in 11%, the commonest surgery performed being enucleation with implant in 11% of surgical cases. Rest of the management done are tabulated in Tables 3 and 4.

**TABLE 3:** Modes of treatment received

Treatment	No of patients (n = 28)	Percentage (%)
Chemotherapy	16	57
Chemo and surgery	4	14
Surgery	3	11
EBRT^a^	3	11
Palliative	2	7

^a^ External beam radiotherapy.

**TABLE 4:** Type of surgeries done

Surgical types	No of patients (n = 28)	Percentage (%)
Enucleation	3	11
Exenteration	1	4
Excision biopsy	3	11
Incision biopsy	2	7
Nil	19	68

On further investigation, 17 of our patients (61%) also had simultaneous metastasis to other sites like brain in nine patients, liver in four, lungs in two, spine in one, and pananasal sinus in one patient.

## 3 DISCUSSION

Metastasis to ocular structures can be either intraocular or orbital. Intraocular structures are the most common site of metastasis from distant site. Among which, choroid, being the most vascular structure, is found to be the commonest structure involved with distant metastasis. In our study, intraocular metastasis was seen in 39% of patients. Ferry AP also reported that in their study of 227 cases with ocular metastasis, 196 patients had intraocular, 28 had orbital, and three had optic nerve metastasis. Their study also showed that metastatic cancer is more common than primary ocular carcinoma.^6^ Shields et al also quotes that most metastatic cancer to ocular region, occurs in posterior uveal tract.^7^, ^8^

Choroid is followed by orbit as the next common site of metastatic cancers. Various studies reports 1‐13% of metastasis occurring in orbit.^9^, ^10^, ^11^, ^12^, ^13^, ^14^, ^15^ Researches also show that these lesions account for about 1% to 13% of all orbital tumors.^16^, ^17^, ^18^, ^19^ In our study due to inclusion of both secondary and metastatic cancers, orbit is found to be the commonest site in 43%, followed by choroid in 39%. Other rare sites are conjunctiva and eyelid.

Diminution of vision in 49% was the commonest clinical presentation followed by proptosis in 16% and growth of mass in 14% in our study. Paranasal sinus cancers spread locally to adjacent orbit often presenting as a proptosis of eyeball as an initial manifestation of sinus tumors. Complete loss of vision in four patients in our study signifies the grievous nature of these diseases. Proptosis and/or globe displacement was the commonest finding (63%‐78%) in few studies done in orbital metastasis.^20^, ^21^ Valenzuela et al in their study on orbital metastasis reports diplopia(48%), pain and vision loss as commonest symptoms while proptosis (in 63%), strabismus and decreased visual acuity as commonest signs.^22^

Metastatic and secondary ocular malignancy does not spare any age group. The age group in our study was similar to that of other studies which range from one to 91 years old. Mean age in different studies did not vary much and was around 47 to 62 years.^5^, ^20^, ^21^, ^22^, ^23^ The children in our study had hamatogenic cancers which are seen to infiltrate vitreoretina causing diminution of vision. However, metastatic tumors in ocular regions is found to be common form neuroblastoma and Ewings sarcoma in a study by Goldberg RA.^24^

There was a slight male preponderance (54%) in our study as well as in other studies.^20^, ^21^, ^22^ However, Shields et al in their two studies of orbital and choroidal metastasis had 64% female patients, where they mention that breast cancer, which is the commonest primary site for choroidal metastasis is more common in females and therefore more females presenting with metastasis.^5^, ^23^

Breast carcinoma as the most common source of ocular metastasis ranging from 29% to 53% of cases in various studies.^5^, ^6^, ^8^, ^22^, ^24^, ^25^, ^26^, ^27^, ^28^ It was followed by lungs in around 8% cases while it was the commonest cause in 21% cases in a study done by Shields et al.^8^, ^21^, ^25^, ^26^, ^27^ In our study also lung carcinoma was the commonest tumor metastasizing the eye (in 14%) which was followed by breast carcinoma (in 11%). However in our study due to inclusion of all types of secondary carcinomas including local invasion and hamatogenic malignancies, Non‐Hodgkin lymphoma, and local orbital invasion from sinonasal carcinoma (25% each) were the commonest type of primary malignancies secondarily invading ocular structures. A similar study done by Yan et al from China also reported nasopharangeal carcinoma as the commonest primary tumor invading ocular structures (in 30% cases).^21^ Other primary sites mentioned in various studies were gastrointestinal carcinomas, liver, renal cell carcinoma, prostrate carcinoma, and skin melanoma.^5^, ^8^, ^22^, ^23^, ^25^, ^26^ It is generally thought believed that metastatic tumors which is spread by hamatogenic route should involve bilateral eyes, but to out assurance, it is not the case. In our study, only 32% had bilateral disease while various studies reports 0%.4%,5%, and 18% only.^5^, ^20^, ^21^, ^23^

However, ocular metastasis usually signifies increased morbidity and mortality among the patients because it is frequently associated with simultaneous metastasis to other organs as well. In our study, 61% of patients had metastasis to other sites as well, the most common one being brain followed by liver and lungs. Arepalli et al in their study on choroidal metastasis mention that 92% of their patient had metastasis to other organs too. Yan et al also mention 30% of their patients having disseminated metastasis.^21^

Due to this reason, most of the patients with ocular metastasis are treated with chemotherapy. In our study, 57% received systemic chemotherapy while 14% had combined chemotherapy and surgery. Enucleation for painful blind eye and excision biopsy were the commonest surgery performed (11% each) in our patient while incision biopsy was done in 7% of the patients to confirm the diagnosis.

The limitation of this study is that it is retrospective in nature and the outcome of the treatment could not be recorded as most of the patients did not follow up with us and instead with the treating medical oncologist only. A further prospective study in collaboration with medical oncologist needs to be done to evaluate the final outcome of these patients. Nevertheless, this study provides a base on which further studies can be done as well as it provides vital information to ophthalmologists and oncologist regarding possibility of ocular metastasis as a differential diagnosis while managing patients with various clinical presentations.

## 4 CONCLUSION

Ocular metastasis can display a wide variety of clinical and imaging features and therefore a high degree of suspicion is required for early diagnosis and prompt metastatic workup should be followed. It is often associated with simultaneous metastasis to other parts of the body signifying poor life prognosis. Hence, multidisciplinary approach with clinical and radiation oncologist should be applied while planning treatment for ocular metastasis.

## CONFLICT OF INTEREST

The authors have no conflict of interest.

## AUTHOR CONTRIBUTIONS

All authors had full access to the data in the study and take responsibility for the integrity of the data and the accuracy of the data analysis. *Conceptualization*, P.R.S.; *Methodology*, P.R.S.; *Investigation*, P.R.S., R.S.; *Formal Analysis*, P.R.S.; *Resources*, P.R.S., R.S.; *Writing‐Original Draft*, P.R.S.; *Writing‐Review & Editing*, P.R.S.; *Visualization*, P.R.S.; *Supervision*, R.S.; *Funding Acquisition*, R.S.

## ETHICAL STATEMENT

Approval was obtained for this retrospective study from the Institutional Review Board of Tilganga Institute of Ophthalmology, Kathmandu, Nepal.

## INFORMED CONSENT

Written informed consent was obtained from the patients for photographs and imaging documentations.

## Data Availability

The data that support the findings of this study are available from the corresponding author upon reasonable request.
